# Seoul Virus in Rats (*Rattus norvegicus),* Hyesan, North Korea, 2009–2011

**DOI:** 10.3201/eid1911.130207

**Published:** 2013-11

**Authors:** Lisi Yao, Zhehao Kang, Yongxian Liu, Fenglin Song, Xiaolong Zhang, Xiaomei Cao, Yunshu Zhang, Yu Yang, Xiaohong Sun, Jing Wang, Kongxin Hu, Licheng Liu, Weijun Chen, Lijun Shao, Baoliang Xu, Baolin Wang

**Affiliations:** Chinese Academy of Inspection and Quarantine, Beijing, China (L. Yao, X. Zhang, X. Cao, Y. Yang, X. Sun, J. Wang, K. Hu, B. Xu, B. Wang);; Ryanggang-do Institution of Commodity Entry-Exit Quarantine, Hyesan, North Korea (Z. Kang);; Jilin Entry-Exit Inspection and Quarantine Bureau, Changchun, China (Y. Liu, Y. Zhang, L. Shao);; Liaoning Entry-Exit Inspection and Quarantine Bureau, Dalian, China (F. Song);; Beijing Institute of Genomics, Beijing (L. Liu, W. Chen).

**Keywords:** Seoul virus, Rattus norvegicus, Hyesan, North Korea, viruses, hantavirus

**To the Editor:** Seoul virus (SEOV), a member of the family *Bunyaviridae*, genus *Hantavirus,* is primarily carried by *Rattus norvegicus* rats. Because members of *Rattus* species are widely distributed, SEOV has the potential to cause human disease worldwide. It has been reported that SEOV causes a milder form of hemorrhagic fever with renal syndrome than Hantaan virus and Dobrava-Belgrade virus and is responsible for 25% of cases of hemorrhagic fever with renal syndrome in Asia (*1*). Although it is well known that SEOV is endemic to China (*2*) and South Korea (*3*), little is known about its distribution in North Korea (*4*).

In September 2009, June and September 2010, and September 2011, a total of 89 *R. norvegicus* rats were trapped in the city of Hyesan (128°30′E, 41°30′N) during the operation of a cooperative rodent surveillance program of China and North Korea. The captured rodents were euthanized with barbiturate (100 mg/kg), weighed, measured, classified by sex, and then autopsied. Lung samples were probed for the large segment of SEOV by reverse transcription PCR by using the RT primer P14 (*5*), the primary PCR primers HAN-L-F1 and HAN-L-R1, and the nested PCR primers HAN-L- F2 and HAN-L-R2 (*6*). PCR products were sequenced by using an ABI 3730 sequencer (Applied Biosystems, Foster City, CA, USA).

A high rate of SEOV infection was detected in *R. norvegicus* rats; 15 (16.8%) of 89 rodent samples tested positive for SEOV by reverse transcription PCR. Infection rates at each surveillance time were 26.7% (4/15) in September 2009, 7.5% (3/40) in June 2010, 28.6% (6/21) in September 2010, and 15.4% (2/13) in September 2011. All infected *R. norvegicus* rats were adults; 9 were male and 6 were female. The rate of nucleotide substitution in these 15 SEOV amplicons (330 bp; GenBank accession nos. KC576788–KC576802, JX853574) was calculated by Bayesian Markov chain Monte Carlo analysis using BEAST 1.74 (*7*). The mean substitution rate, calculated by using the uncorrelated lognormal distribution relaxed molecular clock model and a Bayesian skyline model for the large segment of SEOV, was 8.27 × 10^−3^ substitutions/site/year, with a 95% high posterior density interval that ranged from 1.02 × 10^−4^ to 1.79 × 10^−2^. This substitution rate is about 3 times greater than that for middle and small segments (*2*).

Phylogenetic relationships were assessed by using the uncorrelated lognormal distribution relaxed molecular clock model with the SRD06 substitution model (*8*) in BEAST 1.74. The Hantaan virus strain AA57 (GenBank accession no. AB620033) sequence was used as the outgroup. The resulting phylogenetic tree (Figure) showed that SEOV strains in the city of Hyesan shared >97.3% identity and were all clustered in their own lineages, subdivided into 2 co-existing sublineages. Although the geographic distance from Hyesan to northeastern China (e.g., Liaoning Province) is much less than that between northeastern and southeastern China (e.g., Zhejiang Province) or central China (e.g., Hubei Province), the phylogenetic distance between SEOV strains in North Korea and those in each location in China in clade A, calculated by using MEGA5.1 (*9*), was 0.03, but was only 0.01–0.02 between locations in China.

**Figure:**
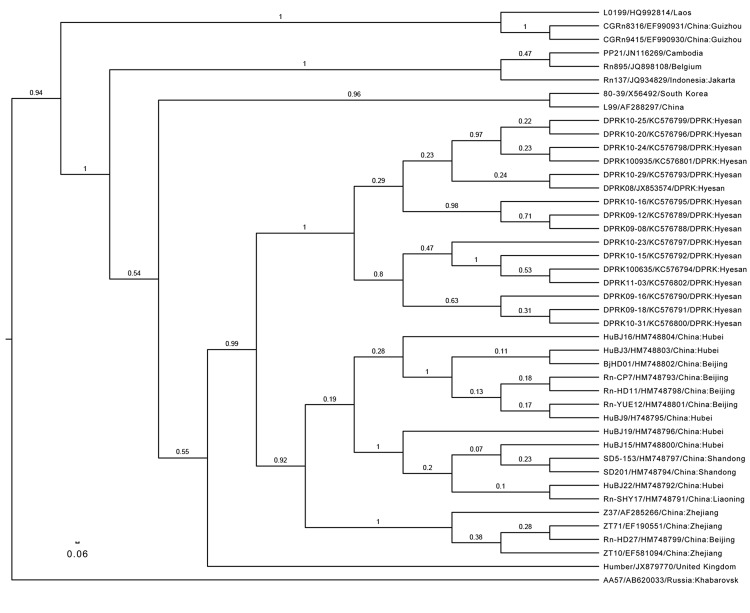
Phylogenetic tree, based on a 330-bp amplicon of the Seoul virus (SEOV) RNA-dependent RNA polymerase gene, depicted in FigTree1.4.0 (http://www.molecularevolution.org/software/phylogenetics/figtree). The tree was generated by using the uncorrelated lognormal distribution relaxed molecular clock model and SRD06 substitution model in BEAST1.74 (*7*). SEOV strain name/GenBank accession no/country: The location is shown in taxa. The posterior number is shown for each branch. Clades A and D were established as described (*2*) Scale bar represents number of nucleotide changes per site.

One possible explanation for this discrepancy in phylogenetic and geographic distances between SEOV strains in China and those in North Korea may be differences in the extent of human contact. Although human interactions among different regions of China are extensive, by comparison, those between China and North Korea are considerably reduced for political reasons. In addition, combining with small segment (GenBank accession no. HQ992815) sequence analysis (data not shown), the fact that SEOV strain L0199 from Laos were not clustered in clade A-D(2) showed that Laos was another possible area of origin for SEOV.

Our work contributes to the known epidemiology of exposure to the SEOV pathogen in Hyesan. Hyesan adjoins Changbai County in Jilin Province of China. However, SEOV was not detected in Changbai County during the surveillance program (data not shown), which was consistent with previous research (*10*). This study further highlights the need for long-term surveillance.
